# Identification and Validation of Potential miRNAs, as Biomarkers for Sepsis and Associated Lung Injury: A Network-Based Approach

**DOI:** 10.3390/genes11111327

**Published:** 2020-11-10

**Authors:** Shaniya Ahmad, Mohd Murshad Ahmed, P. M. Z. Hasan, Archana Sharma, Anwar L. Bilgrami, Kailash Manda, Romana Ishrat, Mansoor Ali Syed

**Affiliations:** 1 Translational Research Lab, Department of Biotechnology, Faculty of Natural Sciences, Jamia Millia Islamia, New Delhi 110025, India; shaniya169053@st.jmi.ac.in (S.A.); archanagmsharma@gmail.com (A.S.); 2 Centre for Interdisciplinary Research in Basic Sciences, Jamia Millia Islamia, New Delhi 110025, India; murshad.60ali@gmail.com; 3 Center of Nanotechnology, King Abdulaziz University, Jeddah KSA.80216, Saudi Arabia; phasan@kau.edu.sa; 4 Deanship of Scientific Research, King Abdulaziz University, Jeddah KSA.80216, Saudi Arabia; bilgrami1956@hotmail.com or; 5 Department of Entomology, Rutgers University, New Brunswick, NJ 08901, USA; 6 Institute of Nuclear Medicine and Applied Sciences, Defense Research Development, Organisation, New Delhi 110054, India; kailashmanda@gmail.com

**Keywords:** sepsis, DEMs, miRNA–mRNA network, CTD, module

## Abstract

Sepsis is a dysregulated immune response disease affecting millions worldwide. Delayed diagnosis, poor prognosis, and disease heterogeneity make its treatment ineffective. miRNAs are imposingly involved in personalized medicine such as therapeutics, due to their high sensitivity and accuracy. Our study aimed to reveal the biomarkers that may be involved in the dysregulated immune response in sepsis and lung injury using a computational approach and in vivo validation studies. A sepsis miRNA Gene Expression Omnibus (GEO) dataset based on the former analysis of blood samples was used to identify differentially expressed miRNAs (DEMs) and associated hub genes. Sepsis-associated genes from the Comparative Toxicogenomics Database (CTD) that overlapped with identified DEM targets were utilized for network construction. In total, 317 genes were found to be regulated by 10 DEMs (three upregulated, namely miR-4634, miR-4638-5p, and miR-4769-5p, and seven downregulated, namely miR-4299, miR-451a, miR181a-2-3p, miR-16-5p, miR-5704, miR-144-3p, and miR-1290). Overall hub genes (*HIP1, GJC1, MDM4, IL6R, and ERC1*) and for miR-16-5p (*SYNRG, TNRC6B, and LAMTOR3*) were identified based on centrality measures (degree, betweenness, and closeness). In vivo validation of miRNAs in lung tissue showed significantly downregulated expression of miR-16-5p corroborating with our computational findings, whereas expression of miR-181a-2-3p and miR-451a were found to be upregulated in contrast to the computational approach. In conclusion, the differential expression pattern of miRNAs and hub genes reported in this study may help to unravel many unexplored regulatory pathways, leading to the identification of critical molecular targets for increased prognosis, diagnosis, and drug efficacy in sepsis and associated organ injuries.

## 1. Introduction

Sepsis is a complex clinical syndrome triggered by the aberrant host response to an infection [1]. It is explicitly lethal as it leads to respiratory failure and ultimately death [2,3]. According to the Global Burden of Disease Sepsis Report, every year, 49 million people are affected resulting in 11 million deaths [4]. The most common infection sites are lungs, urinary tract, abdominal cavity, and primary infection of the circulatory system [5]. Moreover, among all infected sites, the lung is highly vulnerable and the most frequent organ to fail. Accompanying acute lung injury, in sepsis patients, is one of the most captious prognostic markers for mortality [6]. Despite significant advancement in numerous drug development and therapeutic techniques, sepsis is still one of the deadliest emergency department (ED) arrival or nosocomial condition. Delayed diagnosis, disease heterogeneity, genetic variability, and poor understanding of sepsis pathophysiology are the significant causes of poor prognosis. It has become a hidden public health disaster over the past decade [7], and the need for prompt diagnosis and stratification of patient’s accuracy is no less essential [8].

For diagnosis, prognosis, and therapeutic guidance, a large number of biomarkers have been reported including C-reactive protein (CRP) and procalcitonin (PCT) levels, which are used in clinical diagnostic [9]. However, the questionable efficacy of these biomarkers makes them less specific to reflect the pathophysiological changes that occur during sepsis [10,11,12].

miRNAs are non-coding, short (~22 nucleotides) RNAs that bind to the complementary site of target mRNAs, i.e., 3′ untranslated region (UTR), resulting in mRNA degradation with or without translational repression [13,14]. Importantly, they have various biological functions in inflammation, immunity, and different lung injuries [15,16,17]. miRNA pleiotropically target 100–1000 of genes and function in the cell or in an organ-specific manner. One miRNA candidate can regulate the several biological pathways that are perturbed in patients. miRNAs are considered an ideal biomarker as they meets the required criteria such as sensitivity, specificity, and accessibility [18]. miRNAs’ role in sepsis has been investigated by several studies, for instance, a study by Chen et al. demonstrated the altered profiling of specific miRNAs and their targets in patients with sepsis [19]. Previous studies have also demonstrated the correlation of miRNA expression level and mortality of patients with sepsis [20,21]. Several studies have also reported their usage in indicating the presence of pathology, its stage, progression, and genetic link of pathogenesis in different diseases [22,23,24,25,26,27,28]. Furthermore, the altered level of a miRNA may help us in distinguishing disease states, such as in sepsis [29].

A miRNA–mRNA regulatory network can greatly contribute to understanding the pathophysiological process in addition to providing novel and effective therapeutic strategies to treat patients of different lung ailments [30,31,32]. Thus, signature miRNAs and associated hub genes of sepsis disease obtained through our study will provide novel insight into the pathobiology of sepsis behind the perturbation of the disease that can be targeted, and whether these miRNA changes also occur in the lung. We proposed an approach for the interaction between miRNAs and genes by using a network representation, and the identification and utilization of the regulatory networks involved in the pathobiology of sepsis may reveal a new strategy for miRNA gene therapy. Our aim was to determine whether changes in blood miRNA expression in sepsis also affect the expression of miRNAs in the lung microenvironment.

## 2. Materials and Methods

### 2.1. Sepsis-associated Acquisition of miRNA Expression Data

The microRNA expression data series (GSE94717) [33] was obtained from the Gene Expression Omnibus (GEO) database of the National Centre for Biotechnology Information(NCBI) [34]. The series comprises 15 blood samples including patients (*n* = 6) with sepsis-induced acute kidney injury (AKI), patients (*n* = 6) with sepsis non-AKI, and healthy (*n* = 3) controls. This series was based on the Agilent GPL19449 platform (Affymetrix Human Genome U 133 Plus 2.0 110 Array).

### 2.2. Differentially Expressed MicroRNA (DEM) Screening

The expression data were normalized and preprocessed through the GEO2R (http://www.ncbi.nlm.nih.gov/geo/geo2r/) tool to perform DEM analysis between serum samples from sepsis and healthy individuals. GEO2R is a web-based analytical tool that has an in-built Linear Models for Microarray Data (Limma) R package and GEO query. The default parameters were applied for the preprocessing of datasets and DEMs were extracted by applying a cutoff criteria *p* < 0.05 and |log fold-change| > 1.5. Data normalization is a key step to removing technical variation and ensures that meaningful biological comparisons can be made.

### 2.3. Identification of the DEM Target Genes

In the past five years, around twenty-five miRNA target prediction algorithms for mammalian genomes have so far been reported [35]. In our study, we used the following four different databases for target prediction. (1) TargetScan (http://www.targetscan.org/vert_72/) predicts miRNA targets from multiple genomes; this algorithm compares multiple genomes to predict targets [36]. (2) miRmap (https://mirmap.ezlab.org/) is an open-source freely available Python library including web facilities for the prediction of miRNA targets [37]. (3) miRWalk (version 3.0) (http://mirwalk.umm.uni-heidelberg.de/) is a computational approach that is coded in Perl programming language to predict target sites [38]. (4) mirDIP (http://ophid.utoronto.ca/mirDIP/) is a database that provides comprehensive, reliable, and user-friendly resources to predict miRNA targets. A wide range of users use mirDIP, even with little knowledge of statistical analysis or computational approaches [39]. Predicted genes that overlapped in all four databases were considered as target genes. A Venn diagram was generated using the online visualization software VENNY 2.1 (https://bioinfogp.cnb.csic.es/tools/venny/).

### 2.4. Sepsis Gene Extraction from the Database

Sepsis genes were downloaded from the Comparative Toxicogenomics Database (CTD) [40] as disease-related genes. Only sepsis genes were selected, out of 51,000 genes, excluding other diseases (endotoxemia, etc.) from the downloaded data. The CTD database contains almost all published and experimental genes associated with a specific disease. It is a robust, publicly available database that provides manually curated information about gene–disease relationships.

### 2.5. DEM–mRNA Network Construction and Hub Gene Identification

The miRNA–mRNA network was constructed using overlapped genes (target genes vs. CTD sepsis genes) and DEMs in Cytoscape (Version 3.7.1) software and was built manually using SIF files. The Cytoscape plugin cytoHubba (version 0.1) was adopted for identification of the significant modules, subnetwork, and the top-ranked genes/nodes in the given network by several topological algorithms. The hub genes were mapped to corresponding miRNAs using the Cytoscape plugin cytoHubba. The miRNAs having degree cutoff = 2, node score cutoff = 0.2, and *K*-core = 2 were found for each of hub genes. Finally, hub genes and miRNAs were plotted via Cytoscape 3.7.1.

### 2.6. Gene Ontology and Pathway Analysis

To assess the biological implication of identified DEMs, functional enrichment was analyzed in three categories of Gene Ontology, i.e., molecular function, cellular component, and biological processes using DIANA-mirPath. Pathway enrichment analysis was also performed using DIANA- mirPath (a miRNA pathway analysis web server) that offers enriched Kyoto Encyclopedia of Genes and Genomes (KEGG) (https://www.genome.jp/kegg/) pathway visualization.

### 2.7. Experimental Mice Model

A total of eight C57BL/6J mice (8–10 weeks, 20–25 g weight) were procured from an in-house inbred facility of Defence Research Development Organisation (DRDO)-Institute of Nuclear Medicine and Allied Sciences. The study protocols and procedures were approved by the Institute’s animal ethics committee (IAEC) (INM/IAEC/2018/25/ext) and followed as per the guidelines. Mice were caged under ambient condition (temperature of 22–25 °C, 12-h light/dark cycle) with food and water accessibility ad libitum. Mice were employed to establish that the sepsis model underwent the Cecal Ligation and Puncture procedure. To produce sepsis group animals, the mice’s lower abdomen was disinfected and shaved and then an incision was made. Through an incision, the cecum was pulled out and ligated. After ligation, puncture (through and through) was performed using a needle (26-gauge); it was then placed back into the peritoneum and sealed with a non-absorbable 4.0 silk suture (Chromic). Finally, betadine was applied all over the surgery area [41]. Another group of mice underwent a sham operation (same procedure except for puncture and ligation) to produce the control group. All procedures were performed under sterile conditions and mice were kept in sterile cages with food and water availability. Sixteen hours after the operation, the mice were sacrificed and lung tissues were harvested, which were then snap-frozen and stored at −80 °C until RNA isolation was performed [42].

### 2.8. miRNA Expression Validation by Real-Time Quantitative PCR

Total RNA was extracted from lung tissue using Trizol reagent (Ambion, Carlsbad, CA, USA) according to the manufacturer’s protocol and then reverse transcribed into cDNA using an mi-Script (II) RT kit (Qiagen, Germantown, MD, USA). qPCR was conducted using an miScript SYBR Green PCR Kit (Qiagen, Germantown, MD, USA) following the manufacturer’s protocol. Thermocycling conditions for 40 cycles were as follows: initial activation for 95 °C for 5 min, cycle with denaturation 94 °C for 15 s, annealing 55 °C for 30 s, and extension 70 °C for 30 s. RNU6 was used as the internal reference for the miRNA for data normalization and relative expression was calculated by the ΔΔCt method.

### 2.9. Statistical Analysis

Statistical analysis was done using GraphPad Prism version 6 software (San Diego, CA, USA). Data are represented as mean ± SEM. Unpaired *t*-test was applied to analyze the data and Student’s *t*-test was used to analyze real-time gene expression analysis. Statistical significance was taken at 95% confidence interval (*p*-values < 0.05).

## 3. Results

### 3.1. Sepsis-Associated DEM Identification

To identify aberrant miRNAs associated with sepsis, we utilized the GEO dataset GSE94717 [41]. The derived dataset consists of six sepsis-induced AKI, six sepsis non-AKI, and three healthy samples. The dataset contains 2079 miRNAs in total, of which 862 DEMs were identified based on two criteria, i.e., fold change > 1.5 and *p*-value < 0.05. Of these, 11 were upregulated and 851 were downregulated. The expression of the top 10 DEMs in the dataset is presented in Table 1.

### 3.2. Prediction of Target Genes

**Figure 1:**
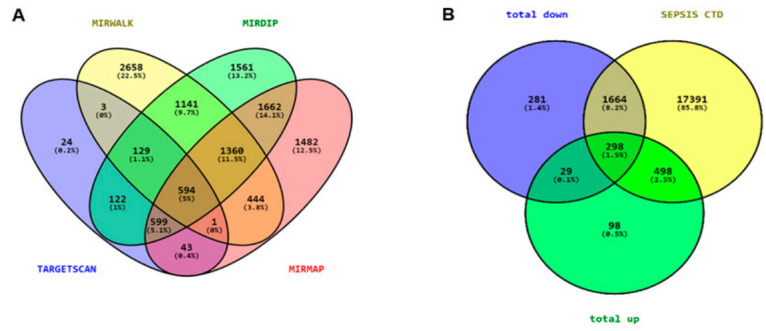
(**A**) Venn diagram for 16-5p showing overlapping genes (594) between four target predictive databases, i.e., TargetScan, miRWalk, mirDIP, and miRmap. For each miRNA, target genes were retrieved using these four databases; each database showed some different target genes, but we extracted the common genes that were validated in all databases. (**B**) Venn diagram showing overlapping genes between target genes and Comparative Toxicogenomics Database (CTD) genes for sepsis. Blue indicates downregulated miRNA target genes and green indicates upregulated miRNA target genes, whereas yellow indicates CTD sepsis genes. In total, 298 genes were found common for all three conditions.

miRNAs exert their regulatory function by binding to the complementary site of target genes resulting into post-transcriptional silencing. To understand the potential role of these top 10 DEMs in the sepsis pathogenesis, we obtained 5773 targets (overlapped genes), as shown in Figure 1A. These target genes were obtained from different databases such as TargetScan, mirMap, mirDIP, and miRWalk. The target genes from the miRWalk, mirMap, TargetScan, and mirDIP databases of the top 10 DEMs were as follows: miR-3074-5p: 4247, 6410, 5531, 13376; miR-1262: 1823, 5290, 4196, 4121; miR-3692-5p: 4816, 6189, 5334, 13023; miR-4299: 3888, 5029, 3903, 12957; miR-4704-5p: 4134, 3641, 2632, 8707; miR-4634: 1526, 880, 639, 4273; miR-323a-3p: 1113, 5421, 489, 2102; miR-4444: 1948, 721, 603, 5961; miR-4638-5p: 4230, 2057, 2027, 12205; and miR-4769-5p: 6589, 5141, 3577, 12236. Overlapped genes obtained from different databases were considered as target genes. In total, 5773 target genes were predicted, of which 4884 were for downregulated miRNAs and 889 were for upregulated miRNAs. For miR-16-5p, 594 genes were found to be common from all four target predictive databases.

### 3.3. Extraction of Disease-Associated Genes and Construction of the DEM–mRNA Network

**Figure 2:**
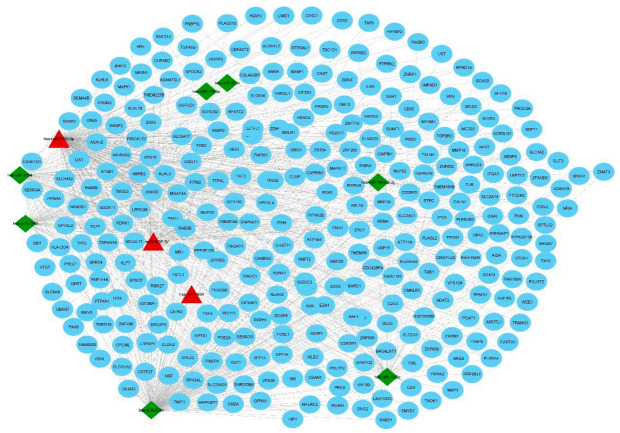
Regulatory network between the top 10 DEMs and their target genes. The target genes were obtained from four different databases and the common ones proceeded forward. This network contains 317 nodes and 763 edges that were constructed using Cytoscape software. Triangles (red) represent upregulated miRNAs, diamonds (green) represent downregulated miRNAs, and circles (blue) represent genes, as an interacting partner.

In total, 17,391 verified sepsis-associated genes (only sepsis genes, excluding other diseases) were obtained from the Comparative Toxicogenomics Database and then cross-referencing with target genes was conducted. In all, 298 overlapping genes were found between target genes (5773) and disease-associated genes (29,161) as shown in the Venn diagram (Figure 1B). The miRNA–mRNA regulatory network was constructed using Cytoscape software (version 3.6.1). We found that the top 10 DEMs (miR-4634, miR-4638-5p, miR-4769-5p, miR-4299, miR-451a, miR-181a-2-3p, miR-16-5p, miR-5704, miR-144-3p, and miR-1290) regulate 317 disease-associated genes (Figure 2). There are 763 edges in the network that represents interactions between miRNAs and these genes.

### 3.4. Module Detection and Pathway Enrichment Analysis

**Figure 3:**
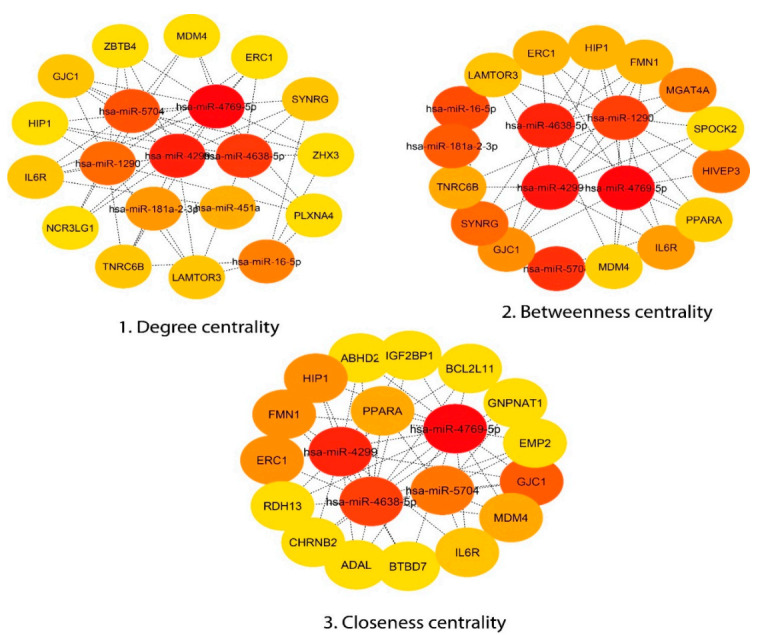
Significant modules and the top 20 ranked genes/proteins of the network based on (**1**) degree, which includes 20 nodes and 52 edges; (**2**) betweenness, which includes 20 nodes and 50 edges; and (**3**) closeness, which includes 20 nodes and 49 edges. The top 20 ranked genes/proteins in the network indicate both gene and miRNA. Red indicates the highest rank, whereas yellow indicates the lowest rank. Based on these modules, 5 hub genes were found to be common in all and considered as significant hub nodes; for miR-16-5p, 3 genes were considered as hub genes.

**Figure 4:**
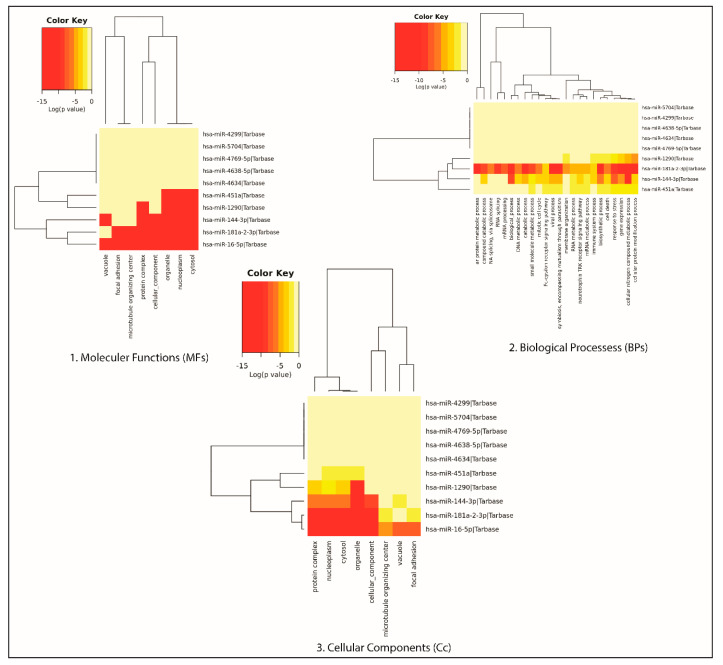
Gene Ontology of the 10 candidate sepsis DEM biomarkers. Representation of functional enrichment of miRs showing (**1**) miRs versus Molecular Functions, (**2**) miRs versus Biological Processes, and (**3**) miRs versus Cellular components, which were retrieved from the DIANA mirPath V3.0 database.

To reduce the complexity and interference of unrelated genes from the list of sepsis-associated genes obtained from the regulatory network, 20 (genes and miRNAs) were identified based on centrality measures, i.e., degree (20 nodes (gene/miR) and 52 edges), betweenness (20 nodes and 50 edges), and closeness (20 nodes and 49 edges) (Figure 3). A total of five common genes (*HIP1, GJC1, MDM4, IL6R, and ERC1*) were considered as hub genes obtained from significant modules (degree, betweenness, and closeness). For miR-16-5p, three genes (*SYNRG, TNRC6B, and LMATOR3*) were considered as hub genes based on two significant modules (degree and betweenness). The color of the top 20 node modules was based on the rank—dark color to light color corresponds to top to bottom rank, respectively. These hub genes were further used for transcription factor (TF) finding, for which we used TRRUST and the Enrichr online databases. These hub genes were also used in the main network to find their interacting partners. However, all five hub genes were connected to miRNAs (Figure 4). The Cytoscape plugin cytoHubba (version 0.1) software was used, which provides a simple interface to analyze a network with 11 scoring methods.

**Figure 5:**
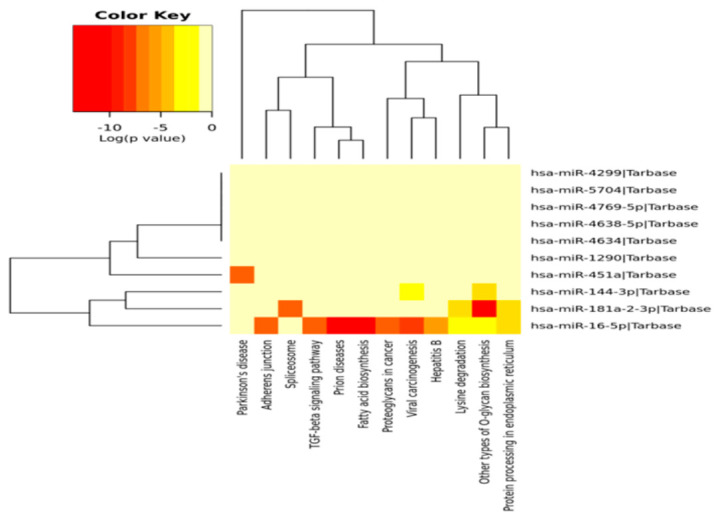
Heatmap of the 10 DEMs (3 upregulated and 7 downregulated miRNAs) showing the top 10 enriched functional pathways (*x*-axis represents the name of the pathways and *y*-axis represents the miRNAs).

Next, pathway enrichment analysis of the 10 DEMs was performed using DIANA-mirPath including KEGG. A heat map was generated via the DIANA-miRPath v3.0 interface of these DEMs (three up and seven down) to show the pathway enrichment analysis (Figure 5). Most of the DEMs were involved in mTOR signaling (7 miR and 25 genes), PI3-Akt signaling (8 miR and 80 genes), and focal adhesion (7 miR and 53 genes). miR-16-5p was involved in the regulation of all these pathways.

### 3.5. Gene Ontology of the DEMs

Functional enrichment was analyzed in three categories of Gene Ontology, i.e., molecular function, cellular component, and biological process using DIANA-mirPath for the 10 DEMs. The DIANA mirPath V3 database (http://amp.pharm.mssm.edu/Enrichr/) was used to perform GO functional annotation. *p*-value < 0.05 was considered as statistically significant. Enrichment analysis is popularly performed to analyze gene sets generated by genome-wide experiments.

The significant biological processes for DEMs are principally associated with the cellular nitrogen compound metabolic process, the cellular protein modification process, and the biosynthetic process. The results showed the enriched molecular function associated with the DEMs. GO analysis also revealed that the genes associated with the DEMs were mainly involved in molecular functions such as ion binding, various enzyme activities, and protein binding during transcription factor-mediated regulation.

The common targeted genes of the 10 DEMs are shown in Table S1, Supplementary Materials.

### 3.6. Validation of miRNA Expression in the Sepsis Model

To confirm the findings’ reliability from the bioinformatics analysis, we validated miRNAs in our well-established sepsis mice model by quantitative real-time PCR. According to the experimental results, the expression of miR-16-5p was found to be downregulated, which is in corroboration with our computational expression analysis. In the sepsis mice model, miR-16-5p was found to be significantly downregulated (*p* < 0.05; Figure 2). Furthermore, in our study, we found that miR-181a and miR-451 were significantly upregulated (*p* < 0.05) in the sepsis model, which did not correlate with our bioinformatics expression analysis.

## 4. Discussion

**Figure 6:**
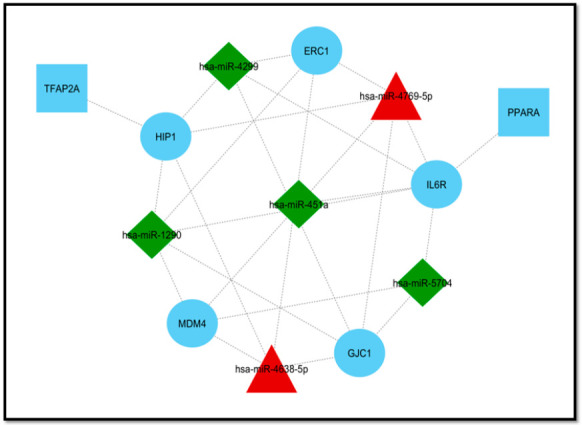
Five hub genes (in blue circles) and their neighbor partner in the main network. Green (diamond) and red (triangle) represent DEMs, and blue (rectangles) represents the transcription factor for the corresponding target hub genes. Transcription factor identification was done from two databases, i.e., TRRUST and Enrichr.

**Figure 7:**
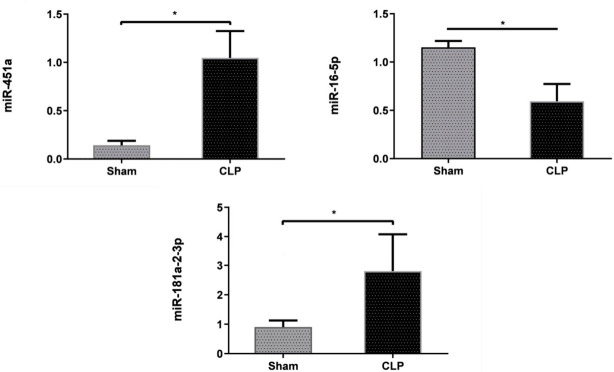
Validation by real-time quantitative PCR of miR-16-5p, miR-451a, and miR-181a-2-3p expressions. Control is indicated by sham group samples. Sepsis is indicated by CLP group samples. Mean ± SEM is represented by the bar, * represents *p* < 0.05, (a minimum of 4 animals were used for the study).

miRNAs play a vital role in cellular signaling and turn to regulate the immune response and molecular pathological outcomes in various sepsis-associated organ injuries including lungs, kidneys, etc. [43,44,45]. Occurrence and progression of sepsis have been associated with dysregulated immune response [46]. Altered miRNAs in platelets and/or macrovesicles were analyzed as potential pathophysiological factors and disease biomarkers in sepsis. miRNA profiling of blood cells (erythrocytes, leukocytes, platelets), serum exosomes, and total serum of sepsis patients showed differences in miRNA expression. The difference in the miR profile was compartment specific. The study pointed out the critical role of exosome-derived miRNAs as sepsis biomarkers for sepsis diagnosis [47]. Similarly, another study also emphasized the role of platelet transcriptome and translational response as potential pathophysiological biomarkers for sepsis patients [48]. Our study aimed to identify the potential differentially expressed miRNA biomarkers and the associated hub genes that may be involved in the dysregulated immune response in sepsis using a comprehensive computational approach. The verified sepsis target genes, which were identified as targets of the 10 DEMs, were used to construct a DEM–gene regulatory network. These 10 DEMs were upregulated (hsa-miR-4634, hsa-miR 4638-5p, and hsa-miR 4769-5p) and downregulated (hsa-miR 4299, hsa-miR 451a, hsa-miR 181a-2-3p, hsa-miR 16-5p, hsa-miR 5704, hsa-miR 144-3p, and hsa-miR 1290). Of the 317 sepsis-associated genes regulated by these DEMs, five (*HIP1, GJC1, MDM4, IL6R, and ERC1*) were designated as hub genes (Figure 6) based on their degrees and other centrality measures (degree, betweenness, and closeness) from the significant modules. The expression of miR-16-5p, 181a-2-3p, and 451a was validated in the lung tissue of the CLP sepsis mice model using the quantitative real-time PCR technique (Figure 7). In vivo expression of miR-16-5p was significantly downregulated, corroborating with our computational findings. Expression of miR-181a-2-3p and miR-451a was found to be upregulated in contrast to our in silico miRNA expression analysis in blood samples of sepsis patients (Figure 7), which suggested different levels of miRNA expressions in blood and lung.

The inflammatory response during sepsis is primarily mediated by the upregulation of the NF-ĸB pathway and the activation of the toll-like receptor (TLR) within the monocytes and macrophages [49]. Consistent with our findings, many researchers have identified miR-16 as a biomarker of sepsis as well as associated lung injury [50,51,52]. Mir-16 downregulation as validated in our study was in line with other studies that reported significant downregulation of miR-16 in a mouse model of lipopolysaccharide (LPS)-induced lung injury [53,54,55,56]. In contrast, elevated miR-16 levels in septic T cells of patients were shown to downregulate NF-κB signaling pathways and CXCL10, leading to the reduced production of pro-inflammatory cytokine in T cells [57,58,59]. Diversity in the expression of miR-16 has been reported among survivors and non-survivors of sepsis. Thus, this may account for the controversial results of miR-16 expression in different study groups by different researchers [60]. Moreover, miR-16 also exerts pro-apoptotic effects in lymphocytes and granulocytes [61]. In contrast to this, TLR4 has been reported as one of the many targets of miR-16, and TLR4-related pathways were targeted by miR-16 to regulate cytokine release and macrophage phagocytosis. Increased levels of anti-inflammatory miR-16 were reported in the sera of surviving sepsis patients as compared to the non-surviving group [60]. In contrast, other studies reported increased miR-16 and miR-451 expression in whole blood of septic mice, and the expression of these miRNAs was not dependent on TLR4, TLR2, or NF-κB-dependent pathways [56,62]. Variations in expressions of miR-16 and miR-451 in red blood cells are usually the prime cause of alterations in the quantified levels of circulating miR-16 and miR-451 [63]. miR-451 regulates ROS generation in macrophages, suggesting that enhanced miR-451 in sepsis-induced lung induces ROS generation in alveolar macrophages [64]. miR-451 regulates a negative cascade via its target YWHAZ protein to modulate pro-inflammatory cytokine expression including TNF, IFN-b, and IL-6 [65]. miRNAs’ expression signature may vary depending on causal organisms (Gram-negative and Gram-positive bacteria); this may thus account for the deviation of results in our validation studies in CLP mice model from the computational analysis. The difference in tissue source used for the quantification of differentially expressed miR may also be the prime cause of the different amount of miR expression obtained in our in vivo validation studies. miR-16 is particularly enriched in the spleen and shows higher expression in non-brain tissues as compared to the brain [66]. For 16-5p, three genes were overlapped in between degree and betweenness (in two modules). These three genes (*SYNRG, TNRC6B, and LAMTOR3*) were considered as hub genes for miR-16-5p. SYNRG (Synergin γ) is known to play a role in membrane trafficking at the trans-Golgi network (TGN) and/or endocytosis. It acts by interacting with the γ subunit of the AP1 clathrin–adaptor complex. Its role has not yet been elaborated in the context of sepsis. TNRC6B (trinucleotide repeat-containing protein 6B), which was found to be another target hub gene of miR-16, is a member of the GW182 family proteins. It is known as the argonaut-interacting protein, a component of siRNA and miRNA-mediated post-transcriptional repression of various target mRNAs through a C-terminal silencing domain [67]. TNRC6B has also been previously associated with a possible role in oxidative stress-related mechanisms and a potential target gene of miR-16 [68]. *LAMTOR3* (*MP1*), another hub gene target of miR-16 in our study, has been previously shown to be upregulated by mutated lipopolysaccharide-binding protein (LBP) [69]. Some studies suggest mitogenic signaling activation for ERK and MEK via LAMTOR3, which is a scaffolding protein [70]. LAMTOR3 is rapidly degraded in a lysosome-independent manner and a proteasome-dependent manner [71].

miR-181 has been designated as a significant inflammatory response marker in whole blood analysis associated with sepsis-induced acute respiratory distress syndrome [72]. Decreased miR-181a expression reduced cell apoptosis of LPS-treated lung epithelial cells by targeting Bcl-2 [73]. Earlier, miR-181a-5p was also reported to be upregulated in RAW 264.7 macrophage cells and the LPS-stimulated sepsis mice model [74]. miR-181b expression was reported to be upregulated in early sepsis and sustained in late sepsis. In vivo blockade of miR-181 after sepsis reduced late-sepsis mortality, improved bacterial clearance, and reduced NFI-A expression, which is responsible for myeloid differentiation during sepsis [75]. The levels of creatine (Cr), alanine aminotransferase (ALT), aspartate aminotransferase (AST), and blood urea nitrogen (BUN) significantly decreased with the miR-181a-5p inhibitor in the serum of mice with sepsis [74]. This was in support of the GO pathway analysis performed in our studies, which also revealed that most of the DEMs in our study were significantly involved in the cellular nitrogen compound metabolic process. Sirtuin-1 (SIRT1) and Importin-α3, a protein critical for NF-κB nuclear translocation, were targeted by miR-181b in vitro and in vivo study models, respectively [76]. Upregulation of miR-181 during sepsis is known to enhance TNF-α mRNA degradation [77] and inhibit LPS-induced inflammation, as indicated by the reduced IL-8 and TNF-α concentrations [78].

Similar to our results, miR-144 has been previously reported as a DEM in sepsis patients as compared to healthy controls, which acts by regulating the NF-kB signaling pathway and NEAT1 in the sepsis model [79,80,81]. No distinct roles and targets have been studied for miR-4634 in sepsis and lung injury, although it has been reported as a DEM in other inflammatory diseases such as rheumatoid arthritis [81]. miR-4638-5p was reported to influence prostate cancer progression via angiogenesis by regulating Kidins220 as well as the downstream activity of the PI3K/AKT and VEGF pathways [82]. miR-1290 has been suggested for reducing inflammatory lung injury by suppressing myosin light chain kinase (MYLK) expression in pulmonary artery endothelial cells following various types of injury [83]. Upregulated miR-5704 has been associated with colon tumors as compared to healthy controls [84]. miR-4769-5p has not yet been explored functionally. It has been reported to be upregulated in breast cancer, but its mechanistic pathways remain to be elucidated [85].

Our study identified five hub genes, i.e., *HIP1*, *GJC1*, *MDM4*, *IL6R*, and *ERC1*, from significant modules. Hsa-miR-16-5p was common in two modules based on degree and betweenness, and the huntingtin-interacting protein 1 (HIP1) was targeted by miR-4299, miR-4769, miR-4638, and miR-1290 in our study. However, no study is available on exploring its role in sepsis-induced inflammation or organ injury. Gap junction γ-1 protein (GJC1), also known as connexin 45 (Cx45) or gap junction α-7 protein (GJA7), is a protein component of gap junction that is encoded by the GJC1 gene. Altered expression of gap junction and cardiac structure proteins were reported during in vivo CLP sepsis. Connexin45 mRNA expression was reduced upon LPS induction of sepsis. It is expressed in the retina and also known as an essential protein for the development of the cardiovascular system, whose defects lead to embryonic lethality [86].

The mouse double minute 4 (MDM4) is known as an important regulator of the tumor suppressor p53. During development, it facilitates MDM2′s E3 ligase activity toward p53 and also restricts p53 activity. MDM4 function as a critical molecule for regulating cellular functionality in response to stress [87]. Its role in sepsis has not yet been explored. Severe systemic inflammation upon infection leads to the production of excessive pro-inflammatory cytokines like IL-6, IFNγ, and TNFα [88]. IL6R (interleukin-6 receptor) is a transmembrane receptor of IL-6 that, upon binding to IL-6, triggers the classical IL-6 downstream signaling pathway. Post-transcriptional modulation of IL-6 has been strongly related to the pathogenesis of sepsis. ERC1 is also known as ELKS1 or Rab6IP2. IкB kinase regulatory subunit ERC1 is required for NFкB activation and cytokine production [89]. ELKS1 regulates the transcription of Stxbp2 and Syntaxin 4 via Kdm2b stabilization to exhibit the regulation of mast cell degranulation [90]. The hub genes identified in our study point out the critical role of microRNA-regulated cytokine production pathways in the pathophysiology of sepsis. Further studies are required to elaborate on the regulatory role of DEMs in identified hub gene-mediated pathways during sepsis, which may provide therapeutic aspects to prevent organ injuries due to sepsis (Table 2).

## 5. Conclusions

The results obtained from the study emphasized the role of the microRNA-mediated hub gene regulatory network during sepsis, which could serve as the basis for identifying potential therapeutic targets. miR-mediated regulation would help in improving our understanding of the dysregulated host–response during sepsis and lung injury. The identified hub genes point out the critical role of microRNA-regulated cytokine production pathways in the pathophysiology of sepsis. The differential expression pattern of miR and hub genes reported in the study may help unravel many unexplored regulatory pathways, leading to the identification of critical molecular targets for increased prognosis, diagnosis, and drug efficacy in sepsis and associated organ injuries.

## Figures and Tables

**Table 1:** Top 10 DEMs (7 downregulated and 3 upregulated) on the basis of log fold change and *p*-value. Upregulated miRs are miR-4634, miR-4638-5p, and miR-4769-5p and downregulated miRs are miR-4299, miR-451a, miR-181a-2-3p, miR-16-5p, miR-5704, miR-144-3p, and miR-1290.

ID	Adj. *p*-Value	*p*-Value	logFC	miRNA_ID	Overlapped Genes Obtained by 4 Databases
147614	0.000488	3.6 × 10^−7^	−3.44103	hsa-miR-4299	2090
42866	0.01638	1.52 × 10^−3^	−3.70826	hsa-miR-451a	45
17928	0.006922	8.51 × 10^−5^	−4.70341	hsa-miR-181a-2-3p	309
10967	0.016261	1.46 × 10^−3^	−2.96498	hsa-miR-16-5p	594
169211	0.035412	1.28 × 10^−2^	−2.45418	hsa-miR-5704	930
29802	0.029521	8.59 × 10^−3^	−2.90199	hsa-miR-144-3p	47
168568	0.03527	1.27 × 10^−2^	−2.47463	hsa-miR-1290	869
169214	0.014676	1.13 × 10^−3^	1.71609	hsa-miR-4638-5p	235
169263	0.004767	3.24 × 10^−5^	2.08135	hsa-miR-4634	22
169328	0.014796	1.17 × 10^−3^	1.88279	hsa-miR-4769-5p	632

**Table 2:** Evidence of the miRNA expression profile in a sepsis population with or without lung injury.

miRNA	Disease	Expression	References
miR-16	Sepsis (prognostic predictor)	Upregulated	[91]
	Sepsis (distinguish sepsis/SIRS (Systemic Inflammatory response syndrome) from healthy control	Upregulated	[60]
	LPS-induced acute lung injury	Downregulated	[53,54]
	LPS-induced lung injury (in vivo model and cell line)	Downregulated	[55]
	LPS-induced lung injury (lung tissue and blood samples)	Upregulated	[56]
	Sepsis (biomarker as T-cell-mediated immunoparalysis)	Upregulated	[57]
	Cirrhotic patients with bacterial infection (sepsis)	Upregulated	[61]
	CLP-induced sepsis mice model (blood samples)	Upregulated	[62]
miR-451	CLP-induced sepsis	Upregulation	[62]
miR-181	LPS-induced acute lung injury	Upregulated	[74]
	Sepsis	Upregulated in early sepsis and downregulated in late	[75]
	Acute lung injury (human blood samples)	Upregulated	[72]
	Sepsis	Upregulated	[77]
	Neonatal sepsis and LPS-induced inflammation	Downregulated	[78]

## Supplementary Materials

The following are available online at https://www.mdpi.com/2073-4425/11/11/1327/s1, Table S1: Targets of miRNAs obtained from different databases.
