# Supplementary material for: Identification and Validation of Potential miRNAs, as Biomarkers for Sepsis and Associated Lung Injury: A Network-Based Approach
Source: Genes (Basel). 2020 Nov 10;11(11):1327. doi: 10.3390/genes11111327 (PMC7696689; doi:10.3390/genes11111327)
Supplement: Supplementary file 1 [file genes-11-01327-s001.zip › genes-960326-supplementary.docx]

**Table S1.** Targets of miRNAs obtained from different databases.

| **miRNA IDs** | **Common Targeted Genes** |
| --- | --- |
| has-mir- 4299 | SHISA7, NKIRAS2, LEFTY2, LRRC58, LAMTOR3, TSPAN3, COL4A3BP, FSTL1, PGAP1, ADAL, SLIT3, SUMF1, SNN, ZNF609, ZFAND5, KLHL6, PLA2G15, VTCN1, GNB5, TIMP3, CHST11, GNG2, MARK2, ZBTB4, G2E3, SMYD1, CDAN1, TMEM184B, NFIA, PRKX, FOSL1, CALN1, GM2A, PTP4A1, VPS36, SLC6A8, RPH3AL, PIK3AP1, ERC1, BARD1, MAVS, OPA3, SLC44A2, CIAPIN1, NFATC2, SLCO1A2, TXLNA, BCL2L11, RAPH1, ADAT2, CDC42BPA, NUP62, PLEKHB2, TMEM154, SLC35E1, SCN3B, SLC25A25, RNFT2, CLMP, FAM219B, TOB2, SRXN1, FITM2, NUAK2, KLHL3, ANKS6, PRELP, TPCN1, PIP4K2B, LIX1, KRT80, ROR1, TAF8, PDE2A, EZH1, MKLN1, BMPR2, PXN, HDX, ATG10, SOX13, ARL10, ADM2, RDH13, IMPAD1, PML, SPRY4, ZNF496, ITGA3, MACC1, MMP24, SMG1, TP73, KIAA1549L, PTGDR2, SH2B3, CPT1A, B4GALNT1, DLG5, ZNF266, NHLRC2, ZNF660, PAX5, ITGA2, RCAN1, TMEM56, TAOK1, SLC6A17, DPYSL3, PAX8, ADAMTSL1, SFTPB, PPP1R12B, RAB21, TNRC6B, MAPK1, IGF2BP1, PDPK1, ARHGAP1, SYNRG, ADAM19, B3GAT1, TSPAN18, ZFP14, WASF2, GDF11, TSPAN14, GPR180, TMEM229B, PLAGL2, FAM199X, PPM1H, AMOTL1, SPOCK2, ZNF561, BTBD7, IL6R, YWHAZ, CD2BP2, EIF2S1, CHRNB2, CAMKK2, TMOD2, PSD3, ASAH2, SEMA4B, OPCML, CLDN2, TUB, HLA-DOA, CMKLR1, HPCAL4, SLC1A2, ZNF710, CD4, SEMA3G, NCR3LG1, ST3GAL1, GJC1, HIST2H2BE, NPTX1, ABHD2, SORCS2, HIP1, SKI, CBFA2T2, RUNX1, FKBP5, RBM27, ZNRF1, MR1, ATP11A, CBX5, ARSB, ADAP1, SHROOM4, ZMIZ1, SPTLC2, VPS13A, FGF5, NUFIP2, TTPAL, MGAT4A, MMAA, FMN1, SRGAP3, CEP63, ZHX3, UBXN7, IPO9, EIF2AK1, SSR1, TBC1D1, KLF7, SSBP2, LIMD1, TAB1, CSTF2T, ITIH5, MEF2D |
| has-mir- 451a | VANGL1, IL6R, DPYSL3, ZDHHC23, SPOCK2, MEF2D |
| has-mir- 16-5p | WEE1, PAPPA, CDK5R1, CAPZA2, KLHL18, ZMAT3, SYNRG, SLC2A14, BTRC, SLC2A3, TRANK1, PAQR3, TNRC6B, CLDN2, HPCAL4, CAPRIN1, MAP4, RAF1, PPM1H, SSR1, TXN2, LAMTOR3, TGFBR3, ITGA2, SOX5, EZH1, COL4A3BP, CIAPIN1, FAT3, NAV1, SENP5, PPP1R11, CRISPLD2, AMOTL1, ARRDC4, MACC1, CCNJL, PPP6R3, TAOK1, ATP1B4, PIK3C2A, LRRC58, PHLPP2, POU3F2, SEMA3A, IPO9, PLXNA4, USP15, ZNF652, RNF213, ZHX3, ARHGEF39, RUNX1 |
| hsa-miR- 5704 | AIDA, DEDD2, LRRC58, KLF7, PAQR5, ELMOD3, RNF144A, PTP4A1, WASF2, TMED10, DCAF8, EZH1, OPA3, TMEM229B, PAX5, TCF7, TMEM154, ZNF660, LIX1, FAT3, ZBTB4, ARRDC4, NSF, CPLX2, EMP2, PLEKHA2, TMEM203, MAPK11, ZMAT3, PARM1, PPARA, MDM4, SLC35E1, PAPPA, OGFOD1, LIMD1, MED29, PPP1R12B, PML, PGAP1, MMP24, NCR3LG1, IL6R, GNPNAT1, IGDCC3, B3GAT1, MLEC, GNAL, PRICKLE2, CALN1, UGGT1, KLHL6, ZDHHC23, SYNRG, NAV1, BTRC, RAD1, EIF2AK1, BNIP2, ALDH1L2, AP5B1, KLHL3, GJC1, SRGAP3, NANOS1, H2AFJ, HRK, MAPKBP1, PLXNA4, GNG4, UBXN7, GFRA1, PSD3, RAB6B, CEP63, ZHX3, QPRT, LRPAP1, ATG7 |
| hsa-miR- 181a-2-3p | SENP5, CPT1A, AMOTL1, PRDM2, CSNK1G1, ZNF609, SMG1, GNG4, CBX5, TMEM184B, TNRC6B, PRKX, RAB3B, NFIA, FNBP1L, LAMTOR3, UST,  PXN, PTGDR2, BANF1, HDAC4, TRANK1, PIK3C2A, NAV2, BDNF, HEG1 |
| hsa-miR- 144-3p | MGAT4A, HIVEP3 |
| has-mir- 1290 | YWHAZ, KLHL6, SYNPO2, FAT3, AIDA, RNF144A, NRXN1, PSD3, MMP16, TNRC6B, PPP1R12B, CD4, ANKH, BICD2, CSNK1G1, GM2A, SLC6A6, PGAP1, CHST11, GNAL, IL6R, HS2ST1, KIAA1191, LZTFL1, CSTF2T, HIP1, MAPK1, GFRA1, FMN1, CBX5, PXMP4, TGFBR3, RPRD1A, MDM4, SFTPB, NAV1, SENP5, PITPNB, NCR3LG1, SEMA3A, PLXNA4, PTPRN2, CHIC1, ITGA2, TCF7, FSTL1, CDS2, PLAGL2, TMOD2, PPARA, SASH3, HIVEP3, HP1BP3, SSR1, CAST, PLEKHA2, RAD1, CPT1A, SPOCK2, ZBTB4, GDF11, ZDHHC23, GPR180, ERC1, MKLN1, MYSM1, KIAA1549L, DBT, SLC6A17, PXN, NUFIP2, LAMTOR3, GJC1, MLEC, |
| has-mir- 4769-5p | PRICKLE2, RPRD1A, FNBP1L, SPTLC2, MLEC, PPP6R3, ZNF266, YWHAZ, SH2B3, ADAL, PLEKHB2, EZH1, SHISA7, PPP1R11, SUMF1, SOX13, PLXNA4, PTGDR2, ADM2, CLDN2, TAOK1, SLC2A3, CAPRIN1, VANGL1, PIK3AP1, MEF2D, DCAF8, SCN3B, ERC1, SLC6A6, FGF5, TSPAN3, ADAT2, HIVEP3, MMAA, HIP1, SLC2A14, SLCO1A2, COL4A3BP, GJC1, ITGA3, TBC1D1, GNG2, KIAA1191, IGF2BP1, MDM4, PPM1H, OPA3, CALN1, PML, HP1BP3, RBM27, CIAPIN1, CDC42BPA, CPLX2, VPS36, TGFBR3, RNF144A, PLAGL2, ZNF609, HIST2H2BE, KLHL18, CDAN1, USP15, CSTF2T, TTPAL, LZTFL1, ZBTB4, GNPNAT1, PTP4A1, WEE1, RAB3B, ARHGEF39, NCR3LG1, BDNF, ATP1B4, SASH3, SOX5, SYNPO2, SLC44A2, SNN, KRT80, PIP4K2B, ARSB, SLC25A25, SMYD1, AIDA, CCNJL, EIF2AK1, RUNX1, HPCAL4, ZHX3, CAMKK2, VPS13A, ELMOD3, SPRY4, TSPAN18, CHIC1, MKLN1, ZNF652, IL6R, NFIA, FAT3, NUAK2, IPO9, CAPZA2, NPTX1, FSTL1, SHROOM4, TMED10, PAQR3, SLC6A17, CAST, PHLPP2, BCL2L11, GFRA1, TMEM154, SRGAP3, FMN1, CDS2, SSBP2, TMEM184B, NAV2, OPCML, CHRNB2, B3GAT1, LEFTY2, FOSL1, NAV1, PAX5, ARRDC4, TNRC6B, TMEM56, LIX1, ZNRF1, RPH3AL, PLA2G15, FAM199X, ATG10, PAPPA, HRK, PITPNB, BTBD7, RNFT2, ALDH1L2, CD4, MAPKBP1, TUB, CEP63, TRANK1, SMG1, SORCS2, NHLRC2, TAB1, ATP11A, MACC1, ARL10, UST, MED29, EMP2, ANKS6, CRISPLD2, CDK5R1, SKI, CMKLR1, RDH13, ZMIZ1, H2AFJ, PIK3C2A, CBFA2T2, VTCN1, KIAA1549L, HDX, PGAP1, SLIT3, PPARA, ROR1, CHST11, KLF7, HS2ST1, TXLNA, ANKH, PSD3, FKBP5, RAPH1, DEDD2, NRXN1, B4GALNT1, AMOTL1, MYSM1, PARM1, ADAM19, G2E3, PDE2A, NSF, PAQR5, TIMP3, IMPAD1, GPR180, ABHD2, LRPAP1, PTPRN2, CD2BP2, SEMA3A, ZFAND5, GM2A, NANOS1, ST3GAL1, MAP4, RAB21, ZNF496, LIMD1, FITM2, SRXN1, BICD2, ADAP1, IGDCC3, ASAH2,  ZNF660, POU3F2, BARD1, NUFIP2, TPCN1, SPOCK2, DLG5, PXMP4, GDF11, TXN2, MAPK11, FAM219B, CLMP, ARHGAP1, QPRT, MMP16, TOB2,  MMP24 |
| has-mir- 4638-5p | NUP62, MDM4, DBT, TMOD2, GNB5, RCAN1, BTBD7, MARK2, ZNF561, RAB6B, PRDM2, UGGT1, LAMTOR3, HIP1, BTRC, BNIP2, TSPAN14, TAF8, ZMAT3, ADAL, NFATC2, HLA-DOA, ATG7, ZDHHC23, EMP2, DPYSL3, SENP5, EIF2S1, PRKX, PDPK1, SYNRG, WASF2, SEMA4B, LRRC58, HEG1, CSNK1G1, SFTPB, MAVS, GJC1, BCL2L11, RAB3B, GNPNAT1, GNAL,  ERC1, CBX5, CHRNB2, ITGA2, ADAMTSL1, TCF7, SLC1A2, HDAC4, FMN1, MAPK1, TMEM229B, KLHL3, OGFOD1, SSR1, PAX8, PXN, RAD1, RDH13, TP73, CPT1A, KLHL6, TMEM203, AP5B1, SLC6A8, MR1, RAF1, ITIH5, IGF2BP1, NKIRAS2, ABHD2, PPARA, RNF213, ZNF710, PRELP, GNG4, UBXN7, BMPR2, PPP1R12B, MGAT4A, BANF1, SLC35E1 |
| has-mir- 4634 | SEMA3G, SYNRG, ZFP14, PLEKHA2 |
